# Temporal trends in neonatal outcomes following iatrogenic preterm delivery

**DOI:** 10.1186/1471-2393-11-39

**Published:** 2011-05-25

**Authors:** Sarka Lisonkova, Jennifer A Hutcheon, KS Joseph

**Affiliations:** 1 Department of Obstetrics & Gynaecology, University of British Columbia and the Women's Hospital and Health Centre of British Columbia, Vancouver, Canada; 2 School of Population and Public Health, University of British Columbia, Vancouver, Canada

## Abstract

**Background:**

Preterm birth rates have increased substantially in the recent years mostly due to obstetric intervention. We studied the effects of increasing iatrogenic preterm birth on temporal trends in perinatal mortality and serious neonatal morbidity in the United States.

**Methods:**

We used data on singleton and twin births in the United States, 1995-2005 (n = 36,399,333), to examine trends in stillbirths, neonatal deaths, and serious neonatal morbidity (5-minute Apgar ≤3, assisted ventilation ≥30 min and neonatal seizures). Preterm birth subtypes were identified using an algorithm that categorized live births <37 weeks into iatrogenic preterm births, births following premature rupture of membranes and spontaneous preterm births. Temporal changes were quantified using odds ratios (OR) and 95% confidence intervals (CI).

**Results:**

Among singletons, preterm birth increased from 7.3 to 8.8 per 100 live births from 1995 to 2005, while iatrogenic preterm birth increased from 2.2 to 3.7 per 100 live births. Stillbirth rates declined from 3.4 to 3.0 per 1,000 total births from 1995-96 to 2004-05, and neonatal mortality rates declined from 2.4 to 2.1 per 1,000 live births. Temporal declines in neonatal mortality/morbidity were most pronounced at 34-36 weeks gestation and larger among iatrogenic preterm births (OR = 0.75, CI 0.73-0.77) than among spontaneous preterm births (OR = 0.82, CI 0.80-0.84); P < 0.001. Similar patterns were observed among twins, with some notable differences.

**Conclusion:**

Increases in iatrogenic preterm birth have been accompanied by declines in perinatal mortality. The temporal decline in neonatal mortality/serious neonatal morbidity has been larger among iatrogenic preterm births as compared with spontaneous preterm births.

## Background

Preterm birth is the leading cause of neonatal mortality and morbidity and preterm infants are more likely to experience neurodevelopmental delay and childhood disability. Thus, the recent increase in preterm birth that has been observed in many industrialized countries is a cause for concern. For instance, in the United States, the rate of preterm birth increased by 20% from 10.6% in 1990 to 12.7% in 2007 [1,2], whereas in Canada the rate of preterm birth increased by 18% from 6.6% in 1990 to 7.8% in 2007 [3,4].

Preterm birth can result from many maternal and fetal causes. Three major clinical subtypes of preterm birth can be identified, namely, iatrogenic (medically indicated) preterm birth, spontaneous preterm birth, and preterm birth following premature rupture of membranes. In North America, the recent increases in preterm birth occurred predominantly due to increases in iatrogenic preterm birth at late preterm gestation (34-36 weeks) [5,6]. Other factors in the increase in preterm birth have included changes in maternal characteristics (such as increases in older maternal age) [7-10] and in the frequency of multiple births [11,12].

Infants born following medically indicated preterm birth are at a two-fold higher risk of neonatal mortality as compared with infants born following spontaneous preterm birth [13-15]. If the risks of adverse birth outcomes among the different preterm birth subtypes have remained unchanged, a temporal increase in preterm neonatal mortality could be expected given the recent increase in iatrogenic preterm births. On the other hand, the recent increases in medically indicated preterm birth have followed improvements in fetal surveillance, obstetric care and neonatal care. This could have resulted in differential reductions in neonatal mortality and serious neonatal morbidity among infants born following iatrogenic preterm birth (as compared with those born following spontaneous preterm birth).

Our goal was to estimate temporal trends in iatrogenic and spontaneous preterm birth and to quantify trends in stillbirth, neonatal mortality and serious neonatal morbidity among the different preterm birth subtypes. We hypothesized that, over the past decade, there have been larger declines in neonatal mortality and serious neonatal morbidity among preterm infants born following obstetric intervention, as compared with infants born following spontaneous preterm labour.

## Methods

We used population-based data on singleton and twin births in the United States, 1995-2005, from the National Centre for Health Statistics (NCHS). Information in the NCHS period linked birth and death files, and fetal death files was abstracted from birth certificates of liveborn infants and from fetal and infant death certificates [16,17]. These data files provided gestational age estimates based on menstrual dates and also the clinical estimate of gestation [18]. The menstrual estimate of gestational age was estimated by the NCHS based on the date of the last normal menstrual period, with the day imputed if missing. The clinical estimate of gestation was that provided by the health care provider, without specification of the source (i.e., whether based on clinical examination, ultrasound, etc). For this study we used the latter, more accurate clinical estimate of gestation at birth [19-21]. We excluded infants born before 24 weeks of gestation, and those weighing less than 500 grams in order to avoid potential bias due to variable birth registration at the borderline of viability [22-24], as attitudes toward such registration may have changed over time. We further excluded infants with a missing clinical estimate of gestational age, a gestational age >45 weeks, or missing data on birth weight or mode of delivery. We excluded an additional 10.9% of births in 2004-05 due to missing data on rupture of membranes; these missing data was related to the introduction of the new birth certificate by some states in 2004-05. Sensitivity analyses were carried out to account for this limitation (see below).

Preterm birth was defined as live birth before 37 completed weeks of gestation, and classified into 3 subtypes using a previously published algorithm [6,15]. Since the NCHS files do not contain direct information on preterm birth subtypes, this algorithm used information on premature rupture of membranes, labour induction, etc, to assign the preterm birth subtype in the following sequence [6,15]: 1) preterm birth following premature rupture of membranes for over 12 hours (PROM); 2) iatrogenic preterm birth (preterm birth following labour induction or caesarean delivery without PROM or conditions indicating prior onset of labour); 3) spontaneous preterm birth (all other births). In the absence of labour induction, preterm birth following caesarean delivery that occurred after the onset of labour (indicated by mention of complications such as precipitous labour, prolonged labour, cephalopelvic disproportion or dysfunctional labour) was assigned to the spontaneous preterm birth category.

Neonatal death was defined as death of an infant that occurred within the first 28 days after birth and serious neonatal morbidity was defined as any of the following conditions: a 5-minute Apgar score ≤3, assisted ventilation ≥30 minutes and neonatal seizures. A composite measure including neonatal death or any of the serious neonatal morbidity listed above was used to estimate the overall rate of adverse neonatal outcomes.

We examined potential differences in maternal characteristics between 1995-96 and 2004-05, with respect to age, race (non-hispanic white, non-hispanic black, hispanic, other), marital status (married or common-law vs. other), education (<12 years vs. 12 years or more), smoking during pregnancy (yes/no) and prior live births (yes/no). We also examined infants' gender, gestational age distributions among stillbirths and neonatal deaths, and the birth prevalence of congenital anomalies.

Temporal trends were quantified by contrasting neonatal mortality and neonatal mortality/serious neonatal morbidity between 1995-96 and 2004-05 using odds ratios and 95% confidence intervals (CI). Temporal changes were further examined within gestational age categories 24-27, 28-31, 32-33, and 34-36 weeks. Odds ratios were reported separately for singletons and twins. Differences in the magnitude of the temporal decline in neonatal mortality or neonatal mortality/serious neonatal morbidity between subtypes of preterm birth (e.g., between the odds ratios expressing the temporal declines in neonatal mortality among preterm birth following iatrogenic delivery vs. spontaneous preterm birth) were assessed using a test for heterogeneity of the odds ratios [25]. We also carried out supplementary analyses to assess if our results were affected by the exclusion of infants with congenital anomalies since temporal increases in prenatal diagnosis and pregnancy termination may have influenced neonatal mortality trends. Sensitivity analyses were also carried out to examine if the exclusion of states which introduced a new birth certificate form in 2004 or 2005 affected our results. Finally, supplementary analyses were carried out to examine if there were temporal difference in outcomes among subgroups such as older mothers (≥35 years). Data used in this study were publicly accessible from the National Centre for Health Statistics. All analyses were performed using SAS statistical package version 9.1.3 (SAS Institute Inc., Cary, NC).

## Results

Maternal characteristics changed during the study period: women who delivered in 2004-05 were older, more educated and smoked less during pregnancy compared with women who delivered in 1995-96 (Table 1). The proportion of births to mothers of Hispanic origin, unmarried mothers and mothers with no prior live births increased, while the frequency of congenital anomalies decreased. The rates of stillbirth and neonatal mortality decreased in all gestational age categories among both singletons and twins (Table 1). The largest declines in stillbirth and neonatal mortality rates occurred at late preterm and term gestational ages.

**Table 1:** Maternal and Infant Characteristics Among Singletons and Twins Born at 24 Weeks of Gestation or Later, United States, 1995-96 and 2004-05

Maternal characteristics*	Singletons			Twins		
	
	1995-96	2004-05	Odds ratio (95%CI)	1995-96	2004-05	Odds ratio (95%CI)
No. of women	6,359,866	6,122,022		82,639	101,572	
Age (years) <20	13.3	10.6	0.77 (0.77-0.77)	7.24	5.14	0.70 (0.68-0.71)
20-24	24.8	25.8	1.06 (1.05-1.06)	19.6	18.0	0.90 (0.89-0.92)
25-29	27.5	27.4	0.99 (0.99-1.00)	27.7	26.1	0.92 (0.91-0.92)
30-34	23.0	22.7	0.98 (0.98-0.99)	28.9	29.1	1.01 (1.00-1.03)
35-39	9.72	11.0	1.15 (1.15-1.15)	14.2	16.9	1.23 (1.20-1.25)
40+	1.73	2.45	1.43 (1.42-1.44)	2.44	4.73	1.98 (1.91-2.06)
Race: Non-Hispanic white	66.2	58.9	0.73 (0.73-0.73)	69.2	65.5	0.84 (0.83-0.85)
African American	16.5	15.4	0.93 (0.92-0.93)	18.1	16.8	0.91 (0.90-0.93)
Hispanic	13.3	20.2	1.64 (1.64-1.65)	9.81	13.4	1.42 (1.39-1.45)
Other	4.04	5.56	1.40 (1.39-1.41)	2.89	4.31	1.51 (1.46-1.57)
Maternal education <12 years	20.8	20.6	0.98 (0.98-0.98)	15.3	13.6	0.84 (0.83-0.86)
Smoking during pregnancy	13.8	11.1	0.78 (0.78-0.78)	12.4	9.00	0.70 (0.68-0.71)
Unmarried	32.4	36.8	1.22 (1.21-1.22)	27.9	28.6	1.03 (1.02-1.05)
No prior live births	42.3	40.4	0.93 (0.92-0.93)	21.9	22.4	1.02 (1.01-1.04)
Infant sex (male)	51.2	51.2	1.00 (1.00-1.00)	49.9	50.2	1.01 (1.00-1.02)
Congenital anomalies (yes)	1.59	1.17	0.74 (0.73-0.74)	2.44	1.66	0.69 (0.66-0.72)
Gestational age-specific						
Stillbirths: 24-27 wks	13.4	11.6	0.85 (0.81-0.90)	6.16	4.66	0.74 (0.64-0.87)
28-31	7.78	6.91	0.88 (0.84-0.92)	3.10	1.89	0.60 (0.51-0.70)
32-33	4.19	3.51	0.83 (0.78-0.88)	1.26	0.59	0.47 (0.37-0.59)
34-36	1.21	0.95	0.78 (0.74-0.81)	0.51	0.28	0.54 (0.45-0.63)
> = 37	0.13	0.11	0.81 (0.79-0.84)	0.24	0.15	0.61 (0.49-0.76)
All	0.34	0.30	0.88 (0.86-0.89)	0.80	0.51	0.64 (0.59-0.69)
Neonatal deaths: 24-27 wks	18.5	16.4	0.87 (0.83-0.91)	23.0	18.5	0.76 (0.69-0.83)
28-31	4.11	3.69	0.89 (0.84-0.95)	3.03	2.54	0.83 (0.72-0.97)
32-33	1.89	1.63	0.88 (0.80-0.96)	0.71	0.62	0.86 (0.66-1.12)
34-36	0.61	0.50	0.81 (0.77-0.87)	0.39	0.25	0.65 (0.53-0.78)
> = 37	0.10	0.08	0.76 (0.73-0.79)	0.20	0.11	0.55 (0.43-0.72)
All	0.24	0.21	0.87 (0.85-0.89)	1.17	1.95	0.80 (0.75-0.86)

*Women with missing information excluded: 5-8% for smoking status and 1-5% for congenital anomalies. All proportions based on live births except for stillbirth rates which were based on total births.

All differences in maternal/infant characteristics between 1995-96 and 2004-05 were statistically significant (P < 0.05) except for the differences in infant sex and the proportion of twin births at 32-33 weeks of gestation.

The overall rate of preterm birth increased from 8.4 per 100 live births in 1995 to 10.5 per 100 live births in 2005. Late preterm births (34-36 weeks), which increased from 5.3 per 100 live births in 1995 to 6.7 per 100 live births in 2005, were responsible for most of the increase. Preterm birth rates increased from 7.3 in 1995 to 8.8 per 100 live births in 2005 among singletons (odds ratio = 1.22, 95%CI:1.21-1.23) and from 52.3 to 62.0 per 100 live births among twins (odds ratio = 1.49, 95%CI:1.46-1.52).

**Figure 1:**
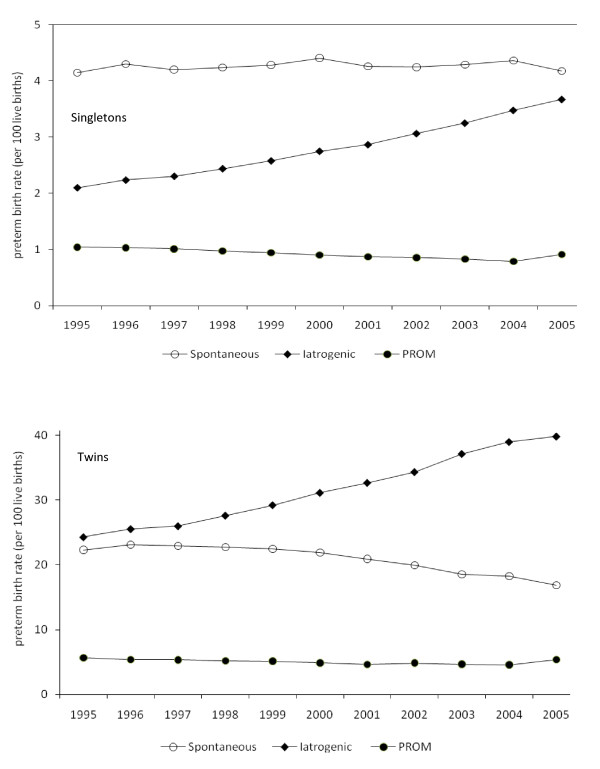
**Preterm birth rates per 100 live births among singletons and twins by subtype of preterm birth, namely, spontaneous preterm birth, iatrogenic preterm birth and preterm birth following premature rupture of membranes for over 12 hours (PROM), United States, 1995 to 2005**.

The increase in the singleton preterm birth rate was predominantly due to an increase in iatrogenic preterm birth from 2.2 in 1995 to 3.7 per 100 live births in 2005 (odds ratio = 1.77, 95%CI:1.76-1.79; Figure 1). The rate of spontaneous preterm birth among singletons was 4.1 per 100 live births in 1995 and 4.2 per 100 live births in 2005, while the rate of PROM preterm birth was 1.0 per 100 live births in 1995 and 0.9 per 100 live births in 2005. Among twins, the iatrogenic preterm birth rate increased from 24.9 to 39.8 per 100 live births from 1995 to 2005 (odds ratio = 2.07, 95%CI:2.03-2.11), while the spontaneous preterm birth rate declined from 21.7 to 16.9 per 100 live births over the same period (odds ratio = 0.70, 95%CI:0.69-0.72; Figure 1). Rates of preterm birth following PROM were 5.7 in 1995 and 5.4 per 100 live births in 2005 (Figure 1).

Neonatal mortality rates declined from 2.5 to 1.9 per 100 preterm live births between 1995-96 and 2004-05 among singletons born following iatrogenic preterm delivery and from 1.6 to 1.2 per 100 preterm live births among singletons born spontaneously. The magnitude of the decline in neonatal mortality following iatrogenic preterm birth (odds ratio = 0.75, 95%CI:0.71-0.78) was not significantly greater than the decline following spontaneous preterm birth (odds ratio = 0.78, 95%CI:0.74-0.81; *P *value for difference in odds ratios = 0.21). There was no significant change in neonatal mortality rates among live births following preterm rupture of membranes between 1995-96 and 2004-05 (odds ratio = 0.95, 95%CI:0.88-1.02). The difference in temporal trends in neonatal mortality between infants born after PROM and those born following iatrogenic and spontaneous preterm birth was significant (*P *value < 0.001 for both contrasts). In general, larger reductions in neonatal mortality were observed at late preterm gestation (34-36 weeks) compared to earlier gestational ages (24-27, 38-31 and 32-33 weeks) (Table 2).

**Table 2:** Temporal Changes in Neonatal Mortality by Plurality, Gestational Age and Subtype of Preterm Birth, United States, 1995-96 and 2004-05

	1995-96		2004-05			
			
Plurality and gestational age and preterm birth subtype	Number of live births	Neonatal deaths/100 live births	Number of live births	Neonatal deaths/100 live births	Odds ratio (2004-05 vs 1995-96)	95% confidence intervals
Singletons						
24-27 wks: Iatrogenic	7,580	17.3	10,202	16.2	0.92	0.85-1.00
Spontaneous	10,295	19.7	8,865	16.5	0.80*	0.75-0.87
PROM	5,410	17.7	4,513	16.8	0.94	0.85-1.05
28-31 wks: Iatrogenic	17,001	4.88	22,023	4.29	0.87*	0.79-0.96
Spontaneous	29,996	3.66	16,913	3.15	0.86*	0.76-0.96
PROM	9,181	3.67	7,351	3.14	0.85	0.72-1.01
32-33 wks: Iatrogenic	17,938	2.55	25,068	2.96	0.76*	0.67-0.87
Spontaneous	26,093	1.62	23,136	1.41	0.87	0.75-1.01
PROM	11,292	1.30	9,075	1.27	0.97	0.76-1.24
34-36 wks: Iatrogenic	101,903	0.88	168,239	0.67	0.75*	0.69-0.82
Spontaneous	206,090	0.48	205,518	0.36	0.75*	0.68-0.82
PROM	40,205	0.55	31,688	0.47	0.85	0.69-1.05
24-36 wks: Iatrogenic	144,422	2.48	225,532	1.86	0.75*	0.71-0.78
Spontaneous	262,474	1.56	254,432	1.22	0.78*	0.74-0.81
PROM	66,088	2.51	52,627	2.38	0.95	0.88-1.02

Twins						
24-27 wks: Iatrogenic	1,953	20.1	3,305	17.7	0.86*	0.74-0.99
Spontaneous	1,955	25.3	1,685	19.8	0.73*	0.62-0.85
PROM	1,035	24.3	1,132	18.7	0.72*	0.59-0.88
28-31 wks: Iatrogenic	4,607	3.30	8,144	2.74	0.83	0.67-1.02
Spontaneous	3,848	2.86	4,060	2.14	0.74*	0.56-0.99
PROM	2,149	2.75	2,333	2.53	0.92	0.64-1.32
32-33 wks: Iatrogenic	6,290	1.95	12,076	0.64	0.67*	0.47-0.94
Spontaneous	5,799	0.47	5,798	0.50	1.07	0.64-1.82
PROM	2,195	0.68	2,575	0.78	1.14	0.58-2.23
34-36 wks: Iatrogenic	29,476	0.42	57,447	0.26	0.63*	0.50-0.80
Spontaneous	24,862	0.32	23,054	0.20	0.64*	0.45-0.92
PROM	3,768	0.58	4,180	0.31	0.53	0.27-1.06
24-36 wks: Iatrogenic	42,326	1.72	80,972	1.28	0.74*	0.67-0.82
Spontaneous	36,464	1.95	34,597	1.43	0.73*	0.66-0.82
PROM	9,147	3.79	10,220	2.97	0.78*	0.66-0.91

*p-value < 0.05

Among twins, neonatal mortality rates between 1995-96 and 2004-05 declined significantly among all 3 preterm birth subtypes. The magnitude of the decline in neonatal mortality among the 3 preterm birth subtypes was similar (*P *> 0.05 for all 3 contrasts). The observed declines in neonatal mortality within each preterm birth subtype were largest among live births at 34-36 weeks gestation (Table 2).

Composite neonatal mortality or serious neonatal morbidity showed a different pattern of change between 1995-96 and 2004-05 (Table 3). The reduction in neonatal mortality/serious neonatal morbidity among singletons was greater among infants born following iatrogenic preterm birth compared with those born following spontaneous preterm birth (odds ratio = 0.75, 95%CI:0.73-0.77 vs. 0.82, 95%CI:0.80-0.84; *P *value for difference in odds ratios <0.001). Neonatal mortality/serious neonatal morbidity rates among preterm live births following PROM increased significantly from 1995-96 to 2004-05 and this change was significantly different from the temporal changes in the 2 other subtypes of preterm birth (*P *value < 0.001 for both contrasts, Table 3). The reductions in neonatal mortality/serious neonatal morbidity in the iatrogenic and spontaneous preterm birth groups were observed at early preterm gestation as well as at late preterm gestation; among live births following preterm PROM, the temporal increase in neonatal mortality/morbidity was observed in the 34-36 week group (Table 3).

**Table 3:** Temporal Changes in Neonatal Mortality or Serious Neonatal Morbidity† by Plurality, Gestational Age and Subtype of Preterm Birth, United States, 1995-96 and 2004-05

	1995-96		2004-05			
			
Plurality and gestational age and preterm birth subtype	Live births	Neonatal outcomes†/100 live births	Live births	Neonatal outcomes†/100 live births	Odds ratio (2004-05 vs 1995-96)	95% confidence intervals
Singletons						
24-27 wks: Iatrogenic	6,418	49.0	7,532	42.9	0.78*	0.73-0.83
Spontaneous	8,569	52.1	6,683	45.5	0.77*	0.72-0.82
PROM	4,728	51.0	3,338	49.9	0.95	0.87-1.04
28-31 wks: Iatrogenic	14,329	28.4	16,008	23.7	0.78*	0.74-0.82
Spontaneous	16,486	23.1	12,501	20.5	0.86*	0.82-0.91
PROM	8,083	26.9	5,402	26.3	0.97	0.89-1.04
32-33 wks: Iatrogenic	14,973	15.8	18,051	13.3	0.82*	0.77-0.87
Spontaneous	21,469	10.7	17,124	9.50	0.88*	0.82-0.94
PROM	9,955	12.3	6,662	12.9	1.06	0.97-1.16
34-36 wks: Iatrogenic	86,586	5.69	119,807	4.49	0.78*	0.75-0.81
Spontaneous	173,286	2.73	152,483	2.43	0.89*	0.85-0.93
PROM	35,844	4.83	22,801	5.64	1.18*	1.09-1.27
24-36 wks: Iatrogenic	122,306	11.9	161,398	9.17	0.75*	0.73-0.77
Spontaneous	219,810	6.95	188,791	5.80	0.82*	0.80-0.84
PROM	58,610	12.9	38,203	13.7	1.07*	1.03-1.12

Twins						
24-27 wks: Iatrogenic	1,640	49.1	2,477	42.0	0.83*	0.73-0.94
Spontaneous	1,671	64.1	1,303	51.7	0.69*	0.60-0.80
PROM	933	59.3	844	55.2	0.85	0.70-1.02
28-31 wks: Iatrogenic	3,875	25.2	5,967	21.6	0.86*	0.78-0.94
Spontaneous	3,312	26.7	3,205	23.7	0.85*	0.76-0.95
PROM	1,889	30.9	1,679	28.5	0.89	0.77-1.03
32-33 wks: Iatrogenic	5,252	12.0	8,930	11.4	0.95	0.85-1.06
Spontaneous	4,981	10.8	4,540	10.9	1.02	0.90-1.16
PROM	1,986	14.7	1,839	13.7	0.92	0.77-1.10
34-36 wks: Iatrogenic	24,983	4.25	42,236	3.65	0.84*	0.78-0.91
Spontaneous	21,432	3.52	18,322	3.47	1.03	0.93-1.14
PROM	3,372	5.60	2,975	6.62	1.19	0.97-1.47
24-36 wks: Iatrogenic	35,750	9.72	59,610	8.20	0.84*	0.81-0.88
Spontaneous	31,396	10.3	27,370	9.36	0.92*	0.87-0.97
PROM	8,180	19.8	7,337	19.0	0.95	0.88-1.03

†Neonatal mortality or serious neonatal morbidity included (neonatal death, 5-minute Apgar score ≤3, neonatal seizures or assisted ventilation for ≥30 minutes).

*p-value < 0.05

Births with missing information about serious neonatal morbidity were excluded. Births recorded on new birth certificates in 2004-05 were excluded due to a different definition of assisted ventilation.

Among twins, neonatal mortality/serious neonatal morbidity rates declined significantly following iatrogenic preterm birth and spontaneous preterm birth, while neonatal mortality/serious neonatal morbidity rates among preterm births following PROM did not change significantly (Table 3). The neonatal mortality/serious neonatal morbidity decrease among iatrogenic preterm births was larger in magnitude than that among spontaneous preterm births (odds ratios = 0.84, 95%CI:0.81-0.88 vs. 0.92, 95%CI:0.87-0.97; *P *value for difference in odds ratios = 0.01). Significant reductions in neonatal mortality/morbidity among twin live births following iatrogenic preterm birth were observed at 24-27, 28-31 and 34-36 weeks but not among those born at 32-33 weeks. Among twins born following spontaneous preterm birth, neonatal mortality/serious neonatal morbidity was significantly reduced at 24-27 and 28-31 weeks but not at 32-33 and 34-36 weeks, while among twins born following PROM, neonatal mortality/morbidity was not significantly reduced in any gestational age group (Table 3).

Supplementary analyses showed that trends in neonatal mortality/serious neonatal morbidity remained unchanged even after the exclusion of infants with congenital anomalies from the analysis. There was a significantly larger temporal decline in neonatal mortality/serious neonatal morbidity following iatrogenic preterm birth (odds ratio = 0.80, 95%CI:0.78-0.82) than following spontaneous preterm birth (odds ratio = 0.92, 95% CI:0.90-0.95; *P *value for difference in odds ratios <0.001). Similar results were also obtained when analysis was restricted to the US states that did not introduce the new birth certificate form in 2004 or 2005. Preterm birth rates were similar between states that did and did not introduce a new birth certificate (10.7 and 10.5 per 100 live births, respectively). Supplementary analyses carried out among older mothers (≥35 years) showed that between 1995-96 and 2004-05, neonatal mortality/serious neonatal morbidity among singletons born preterm following iatrogenic delivery, declined from 10.3 to 7.4 per 100 live births, odds ratio = 0.69 (95%CI: 0.64-0.75); among preterm infants born spontaneously the composite outcome increased from 6.5 to 7.1 per 100 live births, odds ratio = 1.10 (95%CI: 1.03-1.18). Neonatal mortality/serious neonatal morbidity rates remained relatively stable among infants born following PROM (12.7 and 13.2 per 100 live births), odds ratio = 1.05 (95%CI: 0.95-1.16).

## Discussion

We have shown that the temporal increase in preterm birth in the United States between 1995 and 2005 was primarily due to an increase in iatrogenic preterm birth at late preterm gestation among both singletons and twins. This increase in medically indicated preterm birth coincided with reductions in stillbirth rates and neonatal mortality rates. Also, infants born following medically indicated preterm birth showed larger reductions in neonatal mortality and serious neonatal morbidity rates when compared with infants born following spontaneous preterm birth. Neonatal mortality/serious neonatal morbidity rates among infants born following preterm PROM showed a temporal increase among singletons and no significant change among twins.

Our study and previous studies [6,13,14] show that medically indicated preterm birth is the primary cause of the recent increase in preterm birth. This is particularly evident among twins, among whom increases in medically indicated preterm birth have resulted in declines in spontaneous preterm birth. Nevertheless, the various factors responsible for the overall increases in preterm birth (e.g., obstetric intervention, older maternal age and multi-fetal pregnancy) are not mutually exclusive. For instance, population increases in older maternal age lead to increases in medically indicated preterm birth because older maternal age is a risk factor for fetal growth restriction, perinatal mortality and serious neonatal morbidity [26-28].

The reasons for the observed differences in neonatal mortality and serious neonatal morbidity reductions observed among the iatrogenic and spontaneous preterm birth groups probably relate to changes in obstetric surveillance and management. High risk pregnancies with suspected fetal compromise are more carefully monitored currently, with early delivery intervention if the benefits of delivery are deemed to outweigh the risks of preterm birth and expectant management. Given the temporal advances in neonatal care, this effect would be expected mainly at late preterm gestation, when the preterm birth poses less risk to the newborn as compared to earlier gestation. Correspondingly, our findings showed the largest decline in neonatal mortality/serious neonatal morbidity among iatrogenic preterm births which occurred at late preterm gestation. Closer fetal surveillance may also improve outcomes by ensuring that prophylactic antenatal corticosteroid therapy is used, unlike in cases of spontaneous preterm birth where the unexpected onset of labour may preclude such prophylaxis. Maternal transport to a higher level perinatal care facility for labour induction or caesarean delivery may have also contributed to the temporal improvement in neonatal outcomes following iatrogenic delivery. In addition, iatrogenic preterm birth may be carried out for less severe indications in recent years as compared with past years, as the improvements in neonatal care allow for intact survival of preterm infants, especially at late preterm gestation. Although audits of indications for preterm birth show that medically indicated preterm birth is mostly unavoidable and carried out typically for severe or unstable medical/obstetric conditions such as severe preeclampsia or fetal compromise, a small proportion of iatrogenic preterm births may be without a clear medical indication [29,30]. Finally, increases in prenatal diagnosis and pregnancy termination during the study period may have contributed to the differences in trends in neonatal mortality/serious neonatal morbidity following spontaneous and iatrogenic delivery. However, the differences in temporal trends in neonatal mortality/serious neonatal morbidity by preterm birth subtype persisted even after exclusion of infants with congenital anomalies, rendering this explanation unlikely.

The lack of a temporal improvement in neonatal mortality and in neonatal mortality/serious neonatal morbidity among infants born after preterm premature rupture of membranes over 12 hours is concerning. Such infants constitute approximately 1% of singleton live births and approximately 5% of twin live births. Neonatal mortality rates in this preterm category are currently high and the absence of a temporal decline in neonatal mortality/serious neonatal morbidity suggests that this subgroup has not benefitted from recent improvements in obstetric and neonatal care. Research needs to be directed at improving management options for this condition.

The differential temporal reductions in neonatal mortality/serious neonatal morbidity among the iatrogenic and spontaneous preterm subgroups were not evident in contrasts of neonatal mortality. Although reductions in neonatal mortality were somewhat larger among infants born after iatrogenic preterm birth compared with those born following spontaneous preterm birth (odds ratio 0.75 vs. 0.78), this difference was not statistically significant. One possible reason for this may be the lesser frequency of neonatal mortality i.e., the lack of a significant difference could have arisen due to a lesser study power.

The limitations of our study include a potential misclassification of preterm birth subtypes. Some cases of spontaneous preterm labour or PROM who were delivered by caesarean for indications such as fetal compromise may have been misclassified as iatrogenic in our study. This problem arose because our data source did not include details regarding the onset of labour. However, the misclassification introduced because of this is likely small as studies from other more clinically focussed databases (which include information on labour onset) have shown similar proportions of iatrogenic and spontaneous preterm births. For instance, a study from British Columbia, Canada [30], showed that 43% of preterm births in 2005 occurred following preterm labour induction or cesarean delivery in the absence of labour (compared with 42% in 2004-05 in this study). The categorization of preterm birth has been the source of some debate in the past [31,32]. Although each subtype of preterm birth may have a different implication for preventive efforts [33], etiologic pathways are complex and in many instances overlap [31,34].

Another limitation arises because we were not able to utilize data on neonatal morbidity from those states that introduced the new birth certificate forms in 2004 and 2005 due to the incompatibility of definitions for assisted ventilation. However, sensitivity analyses showed that this had a minor impact on our findings. In addition, newborns from states that introduced the new birth certificate during 2004-05 had similar characteristics as compared with infants born in the other states (data not shown). Disease specific information, such as occurrence of intraventricular hemorrhage, necrotizing entrerocolitis or respiratory distress syndrome, was not available in the US data. Instead, we used a composite outcome including neonatal mortality or severe neonatal morbidity, the latter being approximated by Apgar score at 5 minutes < = 3, prolonged ventilation, and neonatal seizures. This composite outcome was chosen to identify neonates who died or those at a high risk of infant death or disability. This composite outcome has been used in previous studies [35] and is strongly associated with adverse outcomes in long-term follow-up studies [36,37]. Data from California, which did not report the clinical estimate of gestation, was excluded from our study (13% of births). This represents a limitation of our study but is balanced by the use of an accurate estimate of gestational age.

## Conclusion

In summary, our study shows that recent increases in obstetric intervention in the United States have resulted in larger declines in rates of neonatal mortality and serious neonatal morbidity among infants born following iatrogenic preterm birth as compared with infants born following spontaneous preterm birth. On the other hand, neonatal mortality/serious neonatal morbidity rates among infants born following PROM showed a temporal increase among singletons and no significant change among twins. Whereas our findings on iatrogenic preterm birth are encouraging, they highlight the need for improving outcomes among preterm infants born following preterm premature rupture of membranes. More research is needed to identify the underlying maternal and fetal conditions that lead to preterm delivery in order to develop targeted interventions to prevent adverse neonatal outcomes resulting from preterm birth.

## Abbreviations

OR: odds ratio; CI: confidence interval; PROM: premature rupture of membranes; NCHS: National Centre for Health Statistics.

## Competing interests

The authors declare that they have no competing interests.

## Authors' contributions

All authors contributed to the conception and design of the study and in the preparation of the manuscript. All authors read and approved the final manuscript.

## Pre-publication history

The pre-publication history for this paper can be accessed here:

http://www.biomedcentral.com/1471-2393/11/39/prepub
